# Psychometric properties of the Japanese version of the career competencies questionnaire for nurses: a cross-sectional study

**DOI:** 10.1186/s12912-022-01035-5

**Published:** 2022-09-27

**Authors:** Masako Yamada, Kyoko Asakura, Nozomu Takada, Yukari Hara, Shoko Sugiyama

**Affiliations:** Tohoku University Graduate School of Medicine, 2-1 Seiryo-machi, Aoba-ku, Sendai, Miyagi Japan

**Keywords:** Career competencies, Career development, Continuous professional development, Nurse, Nursing

## Abstract

**Background:**

Career competencies, which are the knowledge, skills, and abilities essential for career development, have been shown to facilitate career success, fulfilling both work and life goals. In dynamically changing healthcare settings, nurses’ career competencies are key for successfully navigating their careers and improving their nursing practice abilities. However, limited studies have examined career competencies in the nursing profession. In particular, no research has been conducted on career competencies among Japanese nurses, which remains a major challenge as voluntary effort is the main factor promoting career and professional development. Therefore, the purpose of this study was to evaluate the validity and reliability of the Japanese version of the Career Competencies Questionnaire (CCQ-J).

**Methods:**

In this cross-sectional study conducted between June 2020 and August 2021, the English CCQ was translated into Japanese using back and forward translation. Exploratory factor analysis (EFA) and confirmatory factor analysis (CFA) were conducted on separate samples. In the first step, item analysis and EFA were conducted with 276 nurses from one hospital. In the second step, CFA was conducted and concurrent validity and reliability were evaluated with 522 nurses from hospitals in the Tohoku region.

**Results:**

Content validity was confirmed by the back-translation report, an expert panel, and a pilot test. The EFA showed that the CCQ-J consisted of a three-factor structure that explained 66.69% of the variance. The CFA revealed that all the fit indices were acceptable [chi-square value (CMIN) = 432.26, degree of freedom (df) = 153, chi-square fit statistic/degree of freedom (CMIN/df) = 2.83, goodness-of-fit index (GFI) = 0.93, adjusted goodness of fit index (AGFI) = 0.89, comparative fix index (CFI) = 0.96, and root mean square error of approximation (RMSEA) = 0.06]. Cronbach’s α for the 21-item CCQ-J and its subscales ranged from 0.85 to 0.95. Concurrent validity was demonstrated via the positive correlation between work engagement, life satisfaction, and the CCQ-J.

**Conclusions:**

The CCQ-J is a valid and reliable instrument to assess the career competencies of Japanese nurses. We hope that the findings presented in this study will contribute to a better understanding of nurses’ career competencies and their successful career and professional development in the future.

## Background

For nurses, the ability to navigate one’s career is essential for professional development. Dynamically changing healthcare scenarios have expanded nurses’ roles and work settings [1]. In this context, they must acquire advanced knowledge to further evolve as professionals [2]. To achieve this, over the last few decades, postgraduate courses for nurses have become more advanced. Thereby, nursing has become increasingly specialized, and the career paths for nurses to continue improving their knowledge and skills have diversified. Thus, with more work and professional options, it is more important than ever for them to take control of their careers of their own volition. Therefore, the ability to successfully develop one’s career is key for nurses.

The importance of career competencies for successful career navigation in the dynamically changing labor market has been highlighted. Career competencies are defined as “the knowledge, skills, and abilities central to career development, which can be influenced and developed by the individual” [3]. This concept focuses on behaviors that enable individuals to take responsibility for their careers and achieve continuous self-learning and long-term goals. In addition, it helps individuals craft their own careers and enhance specific job competencies in a dynamic work environment [4, 5]. Moreover, individual career competencies extend to organizational, occupational, and industrial contexts. Some studies claim that career competencies are associated with not just individual competencies but also organizational and occupational competencies, which allows organizations to adapt to dynamic environment changes [4, 6]. Hence, career competencies can be seen as an essential resource for workers to successfully navigate their careers while serving in organizational and occupational contexts.

Previous studies on career competencies have explored the relationships between career outcomes, psychological success, and satisfaction. Career competencies have been positively related with both objective career success, such as salary, promotion, and positive performance appraisals, and subjective career success, such as career satisfaction [7]. Furthermore, career competencies have been demonstrated to be positively related with motivational emotion, such as work engagement, well-being, and life satisfaction [8, 9]. Hence, career competencies are clearly associated with navigating one’s career successfully and fulfilling both work and life goals.

However, globally, there have been limited studies on nurses’ career competencies [10]. In particular, no research has been conducted on career competencies among Japanese nurses, which remains a major challenge as individual volition is the main factor that promotes career and professional development. Many empirical studies on careers in Japanese nursing have used the variable of career maturity. However, career maturity is not ideal for examining career development in adults [11]. In other words, career maturity, achieved through continuous professional development, is not a suitable variable for analyzing nurses’ career development. Therefore, at present, no scale adequately measures the competencies necessary for nurses’ career development.

The Career Competencies Questionnaire (CCQ), developed by Akkermans et al. [3], has been evaluated for validity and reliability in Dutch [3, 8], German [12], Lithuanian [9], and English [13]. In addition, it has been used in empirical studies globally [7, 14–20]. Hence, it would be useful to develop a Japanese version of this tool, which is widely used in other countries, for the purpose of comparison. The purpose of this study, therefore, was to evaluate the validity and reliability of the Japanese version of the CCQ (CCQ-J).

Our study makes two main contributions. First, we extend the literature by evaluating whether the CCQ is applicable to the nursing context. To our knowledge, this is the first study to attempt this. The findings may be highly beneficial for nurses’ careers and professional development and will be extended to the provision of quality nursing care. Second, this study contributes to the evidence that the CCQ is a valid measure that is adaptable across cultures and occupations. The present study is the first to assess the adaptability of the CCQ in the Japanese context, extending the literature on global career competencies.

## Methods

### Study design

This study employed a methodological approach to translate the CCQ into Japanese and validate its psychometric properties using a cross-sectional survey.

### Sample and procedures

The population was all nurses working in hospitals in the Tohoku region, Japan (67,791). We used separate samples for exploratory factor analysis (EFA) and confirmatory factor analysis (CFA). In the first step, for the EFA, the minimum sample size was 210. Since the coronavirus disease 2019 (COVID-19) pandemic placed a strain on the medical system, during the first step, we targeted a single hospital to minimize the burden of participation on the medical organization and nurses; registered nurses working at this hospital, which has more than 300 beds, were included in the EFA. The hospital has 317 registered nurses, 14 nursing assistants, with 19 of the former on long-term leave. In general, larger sample sizes increase the generalizability of the results of an EFA [21, 22]. Pett et al. argued that there should be least 10 to 15 subjects per initial item for factor analysis [23]. Hence, the required sample size was at least 210 nurses.

In the second step, to conduct CFA, the sample size was at least 500. We included 80 hospitals randomly selected from a list of hospitals with more than 100 beds in the Tohoku region and asked them to cooperate in the study. Similar to EFA, the larger the sample size for CFA, the better. DeVellis explained that a sample size of 100 is poor, 200 is normal, 300 is good, 500 is very good, and 1,000 is excellent [24]. Hence, the required sample size in the second step was at least 500 participants.

For both EFA and CFA, we included licensed registered nurses and midwives. Since midwives in Japan must possess a registered nursing license, we included midwives as participants. We excluded licensed practical nurses because their qualifications are different from those of registered nurses. Furthermore, we excluded nursing assistants, students not licensed as registered nurses, and nurses on long-term leave.

### Data collection

In the first step, a paper-and-pencil survey was conducted in June 2020. We explained the contents and methods of the study to the nursing director and sub-director both in writing and orally and obtained their consent. We explained in writing to the participants and verbally as well as in writing to the nursing director that participation in the study was voluntary. In addition, we asked the nursing director and sub-director not to pressure the nurses to participate. Since the COVID-19 pandemic has severely impacted nurses’ work and lives, we included a written statement in the study description honoring their commitment and dedication. A total of 298 copies were distributed, then 282 surveys were collected; of these, six were blank or incomplete and were excluded. Therefore, 276 participants were included in the analysis (collection rate: 94.6%, effective response rate: 92.6%).

In the second step, a web survey was conducted between July and August 2021. We mailed the research request letters to 80 surveyed hospitals selected through random sampling, and 24 hospitals returned consent forms for research cooperation (consent rate: 30%). A total of 50 copies with QR codes were mailed to each hospital that provided consent. One hospital specified that only 22 copies of the survey needed to be mailed. Thus, a total of 1,172 copies were distributed. In total, 536 nurses responded to the web survey, which included nine associate nurses who met the exclusion criteria and three blank or incomplete questionnaires. Consequently, 522 nurses were included in the second survey. Table 1 lists the attributes of the participants (collection rate: 45.7%, effective response rate: 44.5%).

**Table 1:** Participants’ characteristics

	First step (*N* = 276)		Second step (*N* = 522)	
Characteristics	n	%	n	%
Gender				
Women	262	94.9	467	89.5
Men	14	5.1	55	10.5
Age				
Mean ± SD^a^	40.6 ± 9.8		40.7 ± 10.5	
Range	21–65		20–63	
Professional experience (years)				
Mean ± SD^a^	17.6 ± 9.6		18.6 ± 10.3	
Range	1–45		0–42	
Health profession				
Nurse	276	100	510	97.7
Midwife	0	0	12	2.3
Education				
High school	98	35.5	71	13.6
Associate degree	139	50.4	355	68
Bachelor’s degree	34	12.3	72	13.8
Master’s degree	3	1.1	8	1.5
Others	2	0.7	10	1.9
No answer	0	0	6	1.1

^a^*SD* Standard deviation

### Measures

#### Nurse information form

We asked the participants to answer questions concerning the following demographics: gender, age, professional experience (years), health profession, and education.

#### Career competencies

Career competencies were assessed using the preliminary 21-item CCQ-J. The original CCQ consists of six first-order factors, each loaded onto three second-order factors. First, reflective career competencies consist of reflection on motivation (three items) and reflection on qualities (four items). Second, communicative career competencies consist of networking (four items) and self-profiling (three items). Third, behavioral career competencies consist of work exploration (three items) and career control (four items) [3]. The Cronbach’s α for the CCQ in the previous study was 0.90, with the coefficient for each subscale ranging from 0.76 to 0.82 [3]. All items are rated on a five-point Likert-type scale ranging from 1 (completely disagree) to 5 (completely agree), evaluated using the total score on each subscale. The total score for the scale ranges from 21 to 105. Higher scores indicate higher career competencies.

#### Work engagement

Work engagement was assessed using the Japanese version of the Utrecht Work Engagement Scale (UWES-J), translated and validated by Shimazu et al. [25]. Work engagement is defined as “a positive and fulfilling work-related state of mind characterized by vigor, dedication, and absorption” [26]. The original scale developed by Schaufeli et al. [27] has a three-factor structure including “vigor,” “dedication,” and “absorption,” but Shimazu et al. found that the one-factor structure fits well [25, 27]. Therefore, in this study, we analyzed it as a one-factor instrument comprising nine items. The items are rated on a seven-point Likert scale ranging from 0 (never) to 6 (always). Responses are evaluated using the total score, which ranges from 0 to 54. The higher the score, the more vigorous and actively involved the participants are in their work. The Cronbach’s α for the UWES-J was 0.92 in the previous study [25] and 0.95 in this study.

#### Life satisfaction

Life satisfaction was assessed using the Japanese version of the Satisfaction with Life Scale (SWLS-J), translated and validated by Oishi [28]. The SWLS-J is a five-item scale (e.g., “I am satisfied with my life”). The items are rated on a seven-point Likert scale ranging from 1 (strongly disagree) to 7 (strongly agree). Responses are evaluated using the total score, which ranges from 5 to 35. The higher the score, the higher the participants’ satisfaction with life. The Cronbach’s α for the SWLS-J in the previous study was 0.76 [28] and in this study was 0.93.

### Translation

The International Society for Pharmacoeconomics and Outcomes Research (ISPOR) guidelines were adopted to translate the English version of the CCQ into Japanese [29]. Before beginning, we obtained permission for translation from Dr. Akkermans, who developed the original Dutch version, and confirmed that it was possible to translate an English version into Japanese. The English version of the CCQ was translated into Japanese by an English and Japanese bilingual translator. The draft scale was back translated by two other translators not involved in the original translation. The draft scale was translated into English by a translator who did not see the original text and prepared a back-translated manuscript. Another translator compared the back-translated manuscript with the original English version and checked it for differences in terminology, expressions, and minor nuances to verify its accuracy. Content validity was confirmed by the back-translation report, which showed no differences in meaning or wording between the back-translation by the translator and the English version of the CCQ items. Since the back-translation report suggested a Japanese translation that more clearly expressed the meaning of the items’ original text and acknowledged the natural flow of Japanese, we accordingly modified the Japanese wording of the draft scale. Additionally, the content validity of the CCQ-J items was confirmed by consensus in an expert panel that included five scholars with doctoral degrees in nursing. To confirm face validity, we conducted a pilot test. We asked four registered nurses to answer the corrected draft scale and check whether any items were difficult to understand. Finally, we reviewed all the items and developed a preliminary CCQ-J.

### Data analysis

Statistical analyses were performed using SPSS version 23.0 (IBM Corp., Armonk, NY, USA) and AMOS version 23.0 (IBM Corp., Armonk, NY, USA). Before the EFA, we evaluated the participants’ descriptive statistics. Skewness and kurtosis were calculated to determine normality. We also evaluated the ceiling effect, floor effect, and item-total correlation. Bartlett’s sphericity test was performed for factor analysis compatibility and the Kaiser–Meyer–Olkin (KMO) measure of sampling adequacy was calculated to determine sample validity.

To investigate how many factors actually contribute to the items translated into Japanese, an EFA was conducted [24]. In the EFA, the number of factors was determined using the scree plot, and factor structures were obtained with Promax rotation. We assessed the factor pattern matrix to determine the factor structure. Since we used oblique rotation, we judged that in a Heywood case, the commonality exceeded 1.0. If the pattern loadings exceeded 1.0, it was confirmed that the commonality exceeded 1.0 [23, 30]. Cross-loading judged the item if the pattern loading was at 3.0 or higher in several dimensions. Some researchers argue that weak loadings are those that are <|.30| [21, 31]. From a practical standpoint, we assessed whether the pattern scores for each factor were >|.30|. Furthermore, items with factor pattern scores >|.40| were retained. If we obtained cross-loading items, we assessed the Cronbach’s α for the group of items loaded on a given factor. According to Pett, αs can be used to evaluate a factor’s internal consistency and decide the best placement for an item with strong loading on several factors [23].

To examine whether the model derived in the previous EFA fit the data, we conducted CFA using the data from the second step. In the CFA, structural equation modeling (SEM) was used to evaluate the construct validity of the factor structure provided by the EFA. We examined the chi-square value (CMIN), goodness-of-fit index (GFI), adjusted goodness-of-fit index (AGFI), comparative fit index (CFI), and root mean square error of approximation (RMSEA) as fit indices.

Concurrent validity was determined based on the directionality of the expected relationships and the strength of the observed correlation coefficient at the same time point [24]. There is no Japanese scale criterion for the CCQ-J. To evaluate concurrent validity, the comparison scales were selected based on reports of excellent reliability and validity and because they were believed to have a theoretical relationship with the CCQ [24]. The variables we used were work engagement and life satisfaction. In line with the Job Demands-Resources model (JD-R model), positive relations have been reported between career competencies with work engagement and life satisfaction [8, 9]. Furthermore, the Japanese work engagement and life satisfaction scales have been verified [26, 29]. We hypothesized that career competencies have a positive correlation with work engagement and life satisfaction at the same time. Pearson’s correlation coefficients of the CCQ-J total score and its subscales between work engagement and life satisfaction were examined. The following criteria were adopted to interpret the correlation coefficients: a weak correlation was indicated by values between 0.1 and 0.3; a moderate correlation by values ≥ 0.3 and < 0.5; and a strong correlation by values ≥ 0.5 [32]. The internal consistency of the full scale and the subscales was examined using Cronbach’s α.

## Results

### Item analysis

The mean and standard deviation of the CCQ-J are presented in Table 2. Kurtosis and skewness did not exceed ± 1. The item-total correlations ranged from 0.52 to 0.81, and the Pearson’s correlation coefficients for all items were significant (*p* < 0.001). In the first and second steps, the sample validity of the KMO for the 21-item CCQ-J was more than 0.9 (first: 0.931, second: 0.939), and Bartlett’s sphericity test was *p* < 0.001, which confirmed the sample validity and goodness-of-fit of the factor analysis.

**Table 2:** Items, means, standard deviations, and pattern matrix of exploratory factor analysis (*N* = 276)

No	Items	Mean	SD^a^	Factor			Communality
				1	2	3	
21	I am able to set goals for myself that I want to achieve in my career	2.87	0.90	**1.023**	-.037	-.167	.83
20	I can create a layout for what I want to achieve in my career	2.80	0.91	**1.002**	-.010	-.123	.88
19	I know what I want to have achieved in my career a year from now	2.90	0.95	**.889**	.130	-.171	.76
18	I can make clear career plans	2.71	0.81	**.876**	-.088	.034	.73
17	I am able to explore my possibilities on the labor market	2.83	0.82	**.663**	-.043	.232	.73
15	I know how to find out what my options are for becoming further educated	2.96	0.84	**.646**	-.008	.260	.83
16	I know how to search for developments in my area of work	3.00	0.83	**.550**	.094	.253	.80
1	I know what I like in my work	3.47	0.80	-.193	**.899**	-.006	.57
4	I know my strengths in my work	3.42	0.79	.005	**.744**	.099	.66
2	I know what is important to me in my career	3.20	0.80	.154	**.720**	-.102	.55
5	I am familiar with my shortcomings in my work	3.72	0.73	-.099	**.671**	.027	.47
3	I can clearly see what my passions are in my work	3.13	0.87	.184	**.653**	-.050	.57
7	I know which skills I possess	3.16	0.80	.174	**.566**	.062	.60
6	I am aware of my talents in my work	2.95	0.81	.062	**.523**	.192	.59
11	I am able to approach the right persons to help me with my career	3.07	0.87	-.075	-.090	**.939**	.65
8	I know a lot of people within my work who can help me with my career	3.10	0.93	-.082	-.010	**.711**	.50
10	I know how to ask for advice from people in my network	3.48	0.76	-.120	.128	**.708**	.57
13	I am able to show others what I want to achieve in my career	2.89	0.87	**.339**	-.055	**.584**	.76
9	I know a lot of people outside of my work who can help me with my career	3.01	0.93	-.087	.127	**.568**	.39
12	I can clearly show others what my strengths are in my work	2.88	0.85	.239	.011	**.566**	.70
14	I can show the people around me what is important to me in my work	2.98	0.87	**.333**	.052	**.497**	.72
	Inter-factor correlations			1	2	3	
	2			.61	―		
	3			.71	.62	―	
	Initial eigenvalues			10.59	1.94	1.48	
	% of variance			50.42	9.23	7.04	
	Cumulative %			50.42	59.65	66.69	

*Note*. Pattern loading > 0.30 is shown in bold

^a^ *SD* Standard deviation

### EFA

**Fig. 1:**
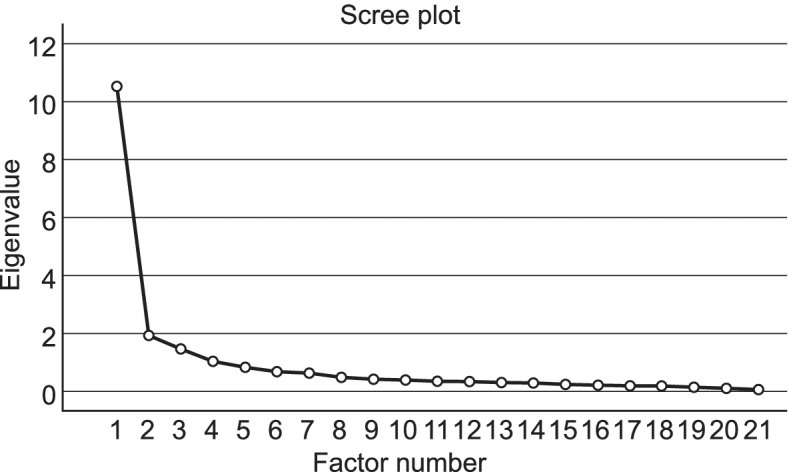
Scree plot of exploratory factor analysis for the Japanese version of the career competencies questionnaire (*N* = 276)

Table 2 shows the results of the EFA and Fig. 1 shows the scree plot. Based on the eigenvalues and scree plot, we determined that the CCQ-J consisted of three factors, accounting for 66.69% of the variance. Compared to the original version, the first, second, and third dimensions reproduced behavioral, reflective, and communicative career competencies. For items 20 and 21, the factor pattern loadings of the first dimension exceeded 1.0, however, the commonality did not exceed 1.0. Cross-loading was identified in items 13 and 14, which loaded at 0.32 or higher on two factors. In Table 2, items 13 and 14 fit with the items in Factor 3, compared with those in Factor 1. Thus, we placed items 13 and 14 in Factor 3 and assessed the inter-item reliability of the two factors with and without items 13 and 14 to determine whether or not these two should be excluded.

### CFA

**Fig. 2:**
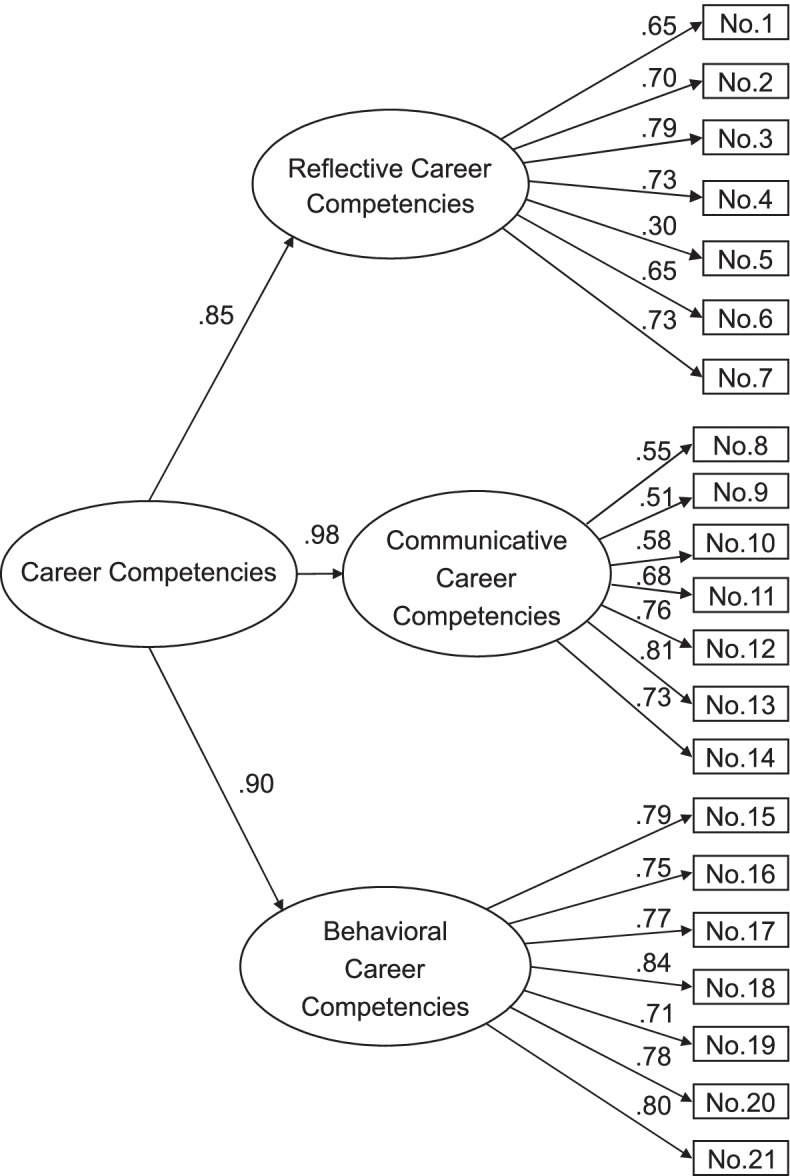
Standardized three-factor structural model of the Japanese version of the Career Competencies Questionnaire (*N* = 522)

To examine whether the three-factor model derived in the preceding EFA fit the data, using data from the second step, we conducted a CFA. The results of the CFA are shown in Fig. 2. The fit indices of the three-factor model were acceptable: CMIN = 432.26 (df = 153, CMIN/df = 2.83), GFI = 0.93, AGFI = 0.89, CFI = 0.96, and RMSEA = 0.06.

### Concurrent validity

The correlations among the CCQ-J in total scores, work engagement, and life satisfaction were *r* = 0.70 and 0.56, respectively (*p* < 0.001). Similarly, a positive correlation was found between the three dimensions of the CCQ-J, work engagement and life satisfaction: *r* = 0.69 and 0.53 for reflective career competencies between work engagement and life satisfaction; *r* = 0.63 and 0.50 for communicative career competencies between work engagement and life satisfaction; and *r* = 0.58 and 0.47 for behavioral career competencies between work engagement and life satisfaction, respectively (*p* < 0.001). The correlation matrix can be found in Table 3.

**Table 3:** Correlations of career competencies with work engagement and life satisfaction and Cronbach’s α (*N* = 522)

	1		2		3		4		5		Cronbach’s α
1. Career competencies											.94
2. Reflective career competencies	.86^***^										.85
3. Communicative career competencies	.91^***^		.68^***^								.85
4. Behavioral career competencies	.91^***^		.64^***^		.75^***^						.92
5. Work engagement	.70^***^		.69^***^		.63^***^		.58^***^				.95
6. Life satisfaction	.56^***^		.53^***^		.50^***^		.47^***^		.65^***^		.93

^***^p < .001

### Reliability

Table 3 shows that the internal consistencies of the overall 21-item CCQ-J and all three dimensions were sufficiently high to conclude that each factor was reliable [33]. Cronbach’s α = 0.94 for the 21-item CCQ-J, α = 0.85 for reflective career competencies (seven items), α = 0.85 for communicative career competencies (seven items), and α = 0.92 for behavioral career competencies (seven items). If items 13 and 14 were excluded, Cronbach’s α = 0.93 for the 19-item CCQ-J, and α = 0.80 for communicative career competencies (five items). In comparison with the 21-item CCQ-J, the Cronbach’s α for the 19-item CCQ-J decreased. Similarly, in comparison with the seven-item communicative career competencies dimension, the Cronbach’s α was lower for the five-item version.

## Discussion

In this study, the English version of the CCQ was translated into Japanese. The reliability and validity of this instrument were subsequently evaluated using a sample of Japanese registered nurses. The CCQ-J was shown to be a reliable and valid scale with a three-factor structure. In the following sections, we discuss the psychometric properties of the CCQ-J, the instrument’s scholarly contribution and implications for practice, and the limitations of this study.

### Psychometric properties of the CCQ-J

Via EFA and CFA, we determined that the CCQ-J consisted of three factors: reflective, communicative, and behavioral career competencies. Surprisingly, this differed from the original factor structure. The original CCQ was a high-order factor model where each of the three factors contained two specific factors. Conversely, in this study, the six first-order factors, that is, the two factors contained in each of the three factors, were not reproduced. This result suggested that the two factors contained in each of the three factors had no clear boundaries. According to Akkermans, the boundaries of “reflection on motivation” and “reflection on qualities” included in reflective career competencies were vague owing to their high factor correlations [3]. In this study, it was possible that these two factors were not clearly distinguishable; therefore, they were likely to be loaded onto one factor. Similarly, communicative career competencies consisted of “networking” and “self-profiling,” while behavioral career competencies consisted of “work exploration” and “career control,” perhaps because of the lack of clear boundaries between these primary factors. This means that the CCQ-J should be treated as the three factor structure in the situation of registered Japanese nurses. Regarding registered Japanese nurses, career competencies may have no particular differences in the two factors contained in each of the three factors.

Our results support that the three dimensions of the CCQ are relevant in the Japanese nursing context. Furthermore, the findings provide evidence that the three dimensions of the CCQ can be used across countries and professions. Globally, studies have adopted the three dimensions of career competencies: reflective, communicative, and behavioral [34, 35]. For example, with the three career competency dimensions in mind, Barnes et al. identified 11 career competencies for academia; these were divided as follows: reflective competencies—gap analysis, self-evaluation, social comparison, and goal orientation; communicative competencies—information seeking and negotiation; and behavioral competencies—strategy alignment, control and agency, university awareness, collaboration, and continuous learning [34]. Since our study also reflects the three dimensions of career competencies, these may be applicable across professions and cultures, which further establishes the validity of the instrument.

In the EFA, items 13 and 14 showed cross-loading and were included in the dimension of communicative career competencies. It can be presumed from the Japanese culture, characterized by modesty, that these items may not be regarded purely as communicative career competencies since they were also loaded onto the dimension of behavioral career competencies of more than 0.3. This could be because East Asians tend toward modesty in self-presentation, even despite a high self-assessment [36]. In Japan, aggressive behavior is not necessarily evaluated when communicating individual strengths, knowledge, skills, and abilities; however, a moderate attitude is highly evaluated. Many previous studies have shown that self-effacing tendencies are often observed in collectivistic social relations in East Asian countries [36–38]. Yamagishi et al. conducted a survey on Japanese and American university students and found that Japanese students exhibited a more self-effacing tendency than Americans when no reason for making an evaluation was presented [36]. Omura et al. conducted a study on Japanese nurses’ assertive communication, finding that the Japanese hierarchical society and cultural virtues of humility and modesty made speaking up almost impossible [39]. Our results that communicative career competencies overlap with behavioral career competencies may also be influenced by the Japanese emphasis on modesty. Thus, it seems correct to presume that these items, included in the dimension of communicative career competencies, may not be suitable for the Japanese culture.

However, we determined that items 13 and 14 need not be deleted. In the analysis of the reliability of the 21-item CCQ-J and the seven items of communicative career competencies, which contained items 13 and 14, Cronbach’s α indicated a sufficient value of 0.8. Conversely, if these items were removed, the reliability of the 19-item CCQ-J and five items of communicative career competencies decreased. Thus, it was valid to include these items in the CCQ-J. Hence, items 13 and 14 were not deleted.

EFA revealed that the configuration of the CCQ-J comprised three factors. Furthermore, CFA showed that career competencies, which were reflective (seven items), communicative (seven items), and behavioral (seven items), were measurable by the total score. Therefore, the CCQ-J had appropriate construct validity.

Reliability was confirmed since the Cronbach’s α was 0.8 or more. However, further verification is required. In this study, reliability was considered only to calculate the α coefficients, and stability verification was not conducted. Hence, it is necessary to verify further reliability, such as using a recert.

Evaluating the concurrent validity of the CCQ-J was based on support for the theoretical relationships, indicating positive correlations between career competencies, work engagement, and life satisfaction at the same time [8, 9]. Our hypotheses in line with the theory of the JD-R model were supported, suggesting parallel validity of the CCQ-J. Future studies should test the theoretical relationships that the CCQ-J has between different variables.

### Contribution to the literature and implications for practice

This study contributes to the advancement of research on career and professional development for Japanese nurses by adapting a scale to measure their career competencies. Measuring career competencies contributes toward conducting research to identify potential issues related to this topic. It allows investigations on whether career barriers negatively affect career competencies, while job resources positively affect them. Future research should investigate the factors that enhance career competencies and their relationship with barriers, thereby clarifying the support required to promote self-directed career and professional development for Japanese nurses. It is useful for nurses to maintain and improve their own nursing practice abilities by applying the knowledge obtained in the empirical study on the CCQ-J. Our results contribute to the provision of quality nursing.

### Limitations

This study has several limitations. First is the use of self-reported data, which contain several potential sources of bias. Second, the psychometric properties of the questionnaire often depend on the characteristics of the sample. The participants in this study were limited to nurses who worked in hospitals in the Tohoku region of Japan. Hence, the results are not generalizable to nurses who work in settings other than hospitals. Therefore, future research should evaluate nurses working in various fields using the CCQ-J adapted in this study. Third, in the COVID-19 pandemic, caution took precedence over research, and to avoid burdening nurses, no test–retest analysis was conducted. Future research should include a test–retest analysis to further strengthen the evidence regarding the validity and reliability of the CCQ-J.

## Conclusions

We evaluated the validity and reliability of the CCQ-J, demonstrating that it consisted of three dimensions: reflective, communicative, and behavioral career competencies. The CCQ-J is an appropriate measure of career competencies that can be used in the context of Japanese nurses. Further studies are required to examine the effectiveness of career competencies with more nurses in various fields, such as home nurses and nursing researchers. We hope that our findings will contribute to a better understanding of nurses’ career competencies and their successful career and professional development.

## Data Availability

The datasets generated and/or analyzed during the current study are not publicly available but can be made available from the corresponding author upon reasonable request.

## Abbreviations

CCQ: Career Competencies Questionnaire
CCQ-J: Japanese version of the Career Competencies Questionnaire
JD-R model: Job Demands-Resource model
UWES-J: The Utrecht Work Engagement Scale
SWLS-J: Japanese version of the Satisfaction with Life Scale
ISPOR: International Society for Pharmacoeconomics and Outcomes Research
KMO: The Kaiser–Meyer–Olkin measure
CMIN: Chi-square value
df: Degree of freedom
CMIN/df: Chi-square fit statistics/degree of freedom
GFI: Goodness-of-fit index
AGFI: Adjusted goodness-of-fit index
CFI: Comparative fix index
RMSEA: Root mean square error of approximation
